# Bacterial-Derived Plant Protection Metabolite 2,4-Diacetylphloroglucinol: Effects on Bacterial Cells at Inhibitory and Subinhibitory Concentrations

**DOI:** 10.3390/biom11010013

**Published:** 2020-12-25

**Authors:** William T. Julian, Anastasia V. Vasilchenko, Daniil D. Shpindyuk, Darya V. Poshvina, Alexey S. Vasilchenko

**Affiliations:** 1 Laboratory of Antimicrobial Resistance, Institute of Environmental and Agricultural Biology (X-BIO), Tyumen State University, 625003 Tyumen, Russia; willjulian09@gmail.com (W.T.J.); vasilchenko.av.83@gmail.com (A.V.V.); shpindyuk-daniil@mail.ru (D.D.S.); dvposhvina@mail.ru (D.V.P.); 2 All-Russian Institute of Plant Protection, 196608 St. Petersburg, Russia

**Keywords:** 2,4-diacetylphloroglucinol, plant protection, membrane permeabilization, subinhibitory effects, quorum sensing, *Pectobacterium carotovorum*

## Abstract

2,4-Diacetylphloroglucinol (2,4-DAPG) is a well-known bacterial secondary metabolite, however, its mechanism of inhibitory and subinhibitory action on bacterial cells is still poorly understood. The mechanism of 2,4-DAPG action on model bacterial strains was investigated using fluorescent spectroscopy and the action of the antibiotic was found to involve a rapid increase in membrane permeability that was accompanied by a reduction in its viability in nutrient-poor medium. At the same time, antibacterial action in nutrient-rich medium developed for several hours. Atomic force microscopy demonstrated time-dependent disturbances in the outer membrane of *Escherichia coli* when exposed to 2,4-DAPG, while *Staphylococcus* *aureus* cells have been visualized with signs of intracellular leakage. In addition, 2,4-DAPG inhibited the metabolic activity of *S. aureus* and *E. coli* bacterial cells in mature biofilms. Observed differences in the antibiofilm activity were dependent upon antibiotic concentration. The intracellular targets of the action of 2,4-DAPG were assessed using bacterial biosensors with inducible bioluminescence corresponding to DNA and protein damage. It was unable to register any positive response from either sensor. As a result, the bactericidal action of 2,4-DAPG is believed to be associated with the destruction of the bacterial barrier structures. The subinhibitory effect of 2,4-diacetylphloroglucinol was tested on quorum-sensing mediated processes in *Pectobacterium carotovorum*. Subinhibitory concentrations of 2,4-DAPG were found to lower the biosynthesis of acyl-homoserine lactones in *P. carotovorum* in a dose-dependent manner. Further investigation elucidated that 2,4-DAPG inhibits the metabolic activity of bacteria without affecting their viability.

## 1. Introduction

Biosecurity is an integrated approach comprising a set of interdisciplinary preventative measures designed to reduce the risk of infectious diseases in humans, animals, and plants [1]. Microbial resistance to antibiotics is an important challenge, but is itself a natural process in which organisms better adapt to changing environmental conditions.

Agricultural losses due to resistant phytopathogens have been evaluated in the billions of dollars. Among the measures taken to stem these losses are the production of novel antimicrobials as well as the implementation of novel approaches to plant biosecurity. The use of live bacterial cells and their metabolites as microbial pest control agents has a long history and has been shown to benefit plants by increasing crop resistance to infection as well as by antagonism of phytopathogens [2]. Direct antagonism of phytopathogens by microbial pest control agents is mediated by secondary metabolites. Secondary metabolites benefit their producers by direct bactericidal action as well as by lowering the virulence of competing organisms through subinhibitory effects. Evaluation of the action of antimicrobial metabolites is critical to the characterization and evaluation of possible microbial pest control agents [3].

2,4-Diacetylphlorglucinol (2,4-DAPG) is a well-known plant protection bacterial metabolite. Numerous studies have demonstrated that *Pseudomonas* spp. producing 2,4-DAPG can suppress a wide variety of plant pathogens, including fungi, bacteria, and nematodes [4]. In addition, there is evidence to presume that 2,4-DAPG has an additional role as a regulatory molecule, as 2,4-DAPG has been shown to impact bacterial and fungal physiology at subinhibitory concentrations [5,6].

Although the antibiotic properties of 2,4-DAPG were recognized over 50 years ago, a detailed mechanism of action has only been described for filamentous fungi and yeasts [7,8,9,10]. Only a preliminary investigation of 2,4-DAPG’s effects on bacteria has been conducted, and this only evaluated 2,4-DAPG’s effects on *S. aureus* [11]. No detailed studies have investigated its effect on planktonic bacterial cells and bacterial biofilms. 

Despite the structural similarities between 2,4-DAPG and known quorum-sensing (QS) inhibitors such as carvacrol and eugenol, the anti-QS properties of DAPG have not been investigated [12]. 

The aim of this work was to study the 2,4-DAPG mode of action on the Gram-positive *Staphylococcus aureus* 209P and Gram-negative *Escherichia coli* K12, which were taken as model strains. In addition, we assessed the activity of 2,4-DAPG on mature biofilms. Subinhibitory effects of 2,4-DAPG also were investigated, specifically by measuring its impact on biosynthesis of QS-autoinducers by *Pectobacterium carotovorum* VKM-B1247.

## 2. Materials and Methods

### 2.1. Bacterial Strains and Purification of Antimicrobial Metabolite

*Pectobacterium carotovorum* VKM-B1247 was obtained from the All-Russian Collection of Microorganisms (Pushchino, Russia). 

2,4-Diacetylphloroglucinol (2,4-DAPG) was obtained by fermentation by *Pseudomonas protegens* CV09183, isolated from the Verevkina cave (Arabica Plateau, Gagra Range, Western Caucasus). 

The bacterium was cultivated in a conical one-liter flask in LB-broth medium (Becton-Dickinson, Sparks, MD, USA) for 24 h. Cells were removed and the medium was prepared for liquid chromatography by one-step purification using a preparative chromatography system (Buchi Reveleris X2, Flawil, Switzerland) equipped with C18-cartridge (Strata C18-E 10 g/60 mL Giga Tubes, 55 µm, 70 Å, Phenomenex, Torrance, CA, USA). Purity of 2,4-DAPG was checked by a high-performance liquid chromatography (HPLC) system equipped with an analytical column (C18, Luna, 250 × 4.6 mm, 5 µm, Phenomenex, Torrance, CA, USA) and diode array detector. The purity of 2,4-DAPG was 95%.

### 2.2. Determination of Minimal Bactericidal Concentration 

Determination of minimal bactericidal concentration was performed using cation-adjusted Mueller-Hinton II broth (MHB; Becton-Dickinson, Sparks, MD, USA). Bacteria at 10^6^ CFU/mL were incubated in 96-well microtiter plates (cat. no. 0030730011, Eppendorf, Hamburg, Germany) containing growth media and various concentrations of 2,4-DAPG in series of two-fold dilutions. The dynamics of bacterial growth were assessed by reading and plotting the absorbance data at 620 nm obtained by the spectrophotometer Multiscan GO (Thermo Scientific, Waltham, MA, USA). Wells without visible bacterial growth were plated on MHB-agar and incubated. Antimicrobial activity was expressed by the minimal bactericidal concentration (MBC), and minimal inhibitory concentrations (MIC) which was defined as the lowest antibiotic dose at which no visible growth in medium and growth on agar-plate were detected, respectively. 

### 2.3. Time-Kill Assay

Cultures of *Escherichia coli* K12 and *Staphylococcus aureus* 209 P were grown to mid-log phase in Mueller–Hinton II broth (MHB; Becton-Dickinson, Sparks, MD, USA). Time-kill studies were carried out based on guideline M26-A of the CLSI [13,14], using Eppendorf non-treated polystyrene 96-well plates (cat. no. 0030730011, Eppendorf, Hamburg, Germany). The kill kinetics of 2,4-DAPG against bacteria were tested by incubating in MHB an initial inoculum of approximately 10^6^ colony forming units (CFU)/mL with antibiotic concentrations at the 0.5MIC, 1MIC and 2MIC. Viable cell counts were determined after 0, 0.5, 1, 2, 4, 6, 8, and 24 h of incubation at 37 °C by plating serially diluted samples onto Muller-Hinton agar plates. Bactericidal activity was defined as a ≥3-log_10_ CFU/mL decrease, in comparison with the baseline, after 24 h of incubation. Indolicidin was used as a positive control, with drug concentrations at MIC and 2MIC. 

### 2.4. Permeabilization of the Cell Wall and Plasma Membrane

This assay was preformed as described previously [15,16]. Membrane integrity was assessed using the fluorescent probes Syto 9 and propidium iodide (PI) (LIVE/DEAD BacLight Bacterial Viability Kit, Molecular Probes, Waltham, MA, USA). The test strains *S. aureus* 209 P and *E. coli* K12 were grown in LB-broth medium (Becton-Dickinson, Sparks, MD, USA) to mid-log phase. The obtained cultures were centrifuged (9391× *g* for 10 min), the supernatant was discarded and the pellet was re-suspended in HEPES buffer (10 mM) to an optical density corresponding to 10^7^ CFU/mL. Bacterial inoculate was transferred to 96-well microtiter plates (cat. no. 0030730011, Eppendorf, Hamburg, Germany) and subsequently supplemented with LIVE/DEAD solution and 2,4-DAPG. Measurements of fluorescence kinetics of Syto 9 were carried out using the plate reader Fluoroscan Ascent FL (Thermo Scientific, Waltham, MA, USA) at 485 nm excitation and 535 nm of emission wavelength. The fluorescence intensity of the samples was estimated in relative fluorescence units (RFU). 

Assessment of bacterial viability in this experiment was performed in parallel series. Bacterial cells have been prepared as described above. Cell’s density was diluted to achieve 10^7^ CFU/mL. 2,4-DAPG taken at various concentrations was mixed with bacteria and incubated for 15, 30, 120, 240, and 480 min. After incubation, bacterial viability was assessed by the drop plate approach.

### 2.5. Microscopy Investigations

#### 2.5.1. Epifluorescence Microscopy

Bacterial cells were grown in LB-broth (Becton-Dickinson, Sparks, MD, USA) to mid-log phase, then centrifuged (9391× *g* for 10 min), re-suspended in HEPES buffer (10 mM) and adjusted to an optical density corresponding to 10^8^ CFU/mL. 2,4-DAPG was then mixed with *E. coli* and *S. aureus* 209P at concentrations corresponding to their MIC. After a 2-h incubation at 25 °C, the cells were washed with distilled water. For microscopy, the suspension was mixed with 0.4% agarose, LIVE/DEAD dyes, and plated. Fluorescence microscopy of stained bacteria was performed using a Zeiss Axio Imager M2 fluorescent microscope (Zeiss, Oberkochen, Germany) equipped with filter sets for simultaneous viewing of Syto 9 and PI fluorescence.

#### 2.5.2. Atomic Force Microscopy

*E. coli* K12 was grown in Mueller–Hinton II broth (Becton-Dickinson, Sparks, MD, USA) to mid-log phase, then centrifuged (9391× *g* for 10 min), re-suspended in HEPES buffer (10 mM) and adjusted to an optical density corresponding to10^8^ CFU/mL. 2,4-DAPG was then added to media containing bacterial cells at final concentrations corresponding to MBC. During incubation, the samples were taken at various time of treatment. Bacterial cells were washed with distilled water, applied to a freshly cleaved mica and dried at 25 °C.

Atomic force microscopy was carried out using “Integra NT-MDT” (NT-MDT, Moscow, Russia) microscope in tapping mode. Microscopy investigation was performed using the cantilever NSG01 (Tipsnano, Tallinn, Estonia) with spring constant ~5.1 N/m. Preliminarily, wide scanning of about 50 × 50 μm^2^ was conducted to produce a reference map of the sample surface so as to help localize the bacterial cells. The scan size was then set with sampling of 512 by 512 points and scan rate of 0.5 Hz. Topographic, amplitude (MAG) and phase images were acquired simultaneously (AFM-images obtained in MAG-mode showed in Figures S1 and S2). 

### 2.6. Antibiofilm Activity of 2,4-DAPG (2,4-Diacetylphloroglucinol)

The assay was performed as previously described with some modifications [17]. The culture of *S. aureus* 209P and *E. coli* K12 were grown in a flat-bottomed 96-well microtiter plates (Eppendorf, Hamburg, Germany) filled with LB-broth (Becton-Dickinson, Sparks, MD, USA). The microplates were incubated at 37 °C for 24 h, after which the media was discarded and fresh media added. After 48 h of incubation the planktonic cells were removed and the plates were gently rinsed twice with 10 mM HEPES buffer. 2,4-DAPG dissolved in HEPES buffer (10 mM) was added to the wells in two-fold serial dilutions up to a final concentrations in range 0–300 µg/mL (*E. coli* K12) or 0–150 µg/mL (*S. aureus* 209P). Microtiter plates were incubated at 25 °C for 3 h. 

After treatment, antibiotic solutions were discarded, and the wells were rinsed twice with HEPES buffer (10 mM). 

Solutions of 2,3,5-triphenyl-tetrazolium chloride (TTC) (DiaM, Moscow, Russia) were prepared by dissolving TTC in distilled water at concentrations of 1% and then sterilized by filtration on 0.22 μm polyvinylidene fluoride (PVDF) filters. TTC solution was added to the biofilms formed in the microtiter wells up to a final concentration 0.25% 100 μL of LB-broth was added to the wells. Plates were incubated in the dark for 3 h at 37 °C. After the incubation period, the well contents were removed, and 95% ethanol was added for 10 minutes. Dissolved formazan was removed to a new flat-bottomed microplate and the absorbance was measured at 490 nm.

### 2.7. Sub-Inhibitory Action of 2,4-DAPG 

#### 2.7.1. SOS-Response and Protein Disturbance

DNA damage and protein denaturation induced in bacteria by 2,4-DAPG were assessed using the *E. coli* MG1655 reporter strains. One of this carries the hybrid plasmids p*RecA* and p*IbpA* in which genes *luxCDABE* of the soil luminescent bacteria *Photorhabdus luminescens* are located under the control of corresponding inducible promoter *recA* (SOS-response)*,* and *ibpA* (heat shock) [18]. 

The bacterial strain was cultivated in LB-broth medium containing ampicillin (100 µg/mL). The overnight culture of the biosensors were washed and re-suspended in pure water to obtain a cell density corresponding to 10^7^ CFU/mL.

Ninety microliters of bacterial suspension were mixed with an appropriate volume of two-fold dilutions of 2,4-DAPG and placed into wells of a 96-wells microplate with non-transparent side walls (Eppendorf, Hamburg, Germany) up to the final volume of 100 μL. Wells filled with sterile deionized water and containing an appropriate amount of bacterial biosensor were used as the negative control. Wells containing biosensor and the 0.25 MIC of norfloxacin (Sigma-Aldrich, Saint Louis, MI, USA) or 9.6% ethanol were used as positive controls. Bioluminescence measurements were carried out using the plate reader Fluoroscan Ascent FL (Thermo Scientific, Waltham, MA, USA) which dynamically registered the luminescence intensity of the samples for 2 h and were estimated in relative light units (RLU). The resulting values of bioluminescence were estimated in induction factor (*R*), which was processed according to the following Equation (1):

*R* = *It*_120 min_ − *It*_0 min_/*Ic*_120 min_ − *Ic*_0 min_ (1)

where *It* is intensity of bioluminescence of a treated sample; *Ic* is intensity of bioluminescence of the control sample (in the absence of inductor).

#### 2.7.2. Quenching of Bacterial Quorum Sensing by 2,4-DAPG

##### Investigation of the QS-Quenching Properties of 2,4-DAPG on *P. carotvorum* VKM-B1247

Bacteria grown overnight were suspended in fresh LB-broth (Becton-Dickinson, Sparks, MD, USA) and incubated at 27 °C. Bacterial growth was monitored in real-time by spectrophotometry every 30 min. After 6.5 h of incubation, 2,4-DAPG was added to growing *P. carotovorum* VKM-B1247. Cells were precipitated by centrifugation (9391× *g* for 15 min) after periods of 3 h from the introduction of 2,4-DAPG, and the supernatants were collected in a separate plate for analysis.

#### 2.7.3. Quantification of Acyl-Homoserine Lactones Using a Bioluminescence-Based Assay

*E. coli* MG 1655 pVFR1-lux is a bioluminescence-based QS-biosensor [19,20]. This strain carries the plasmid pVFR1, which possesses the *luxRI’: luxCDABE (I*’meaning *luxI* mutated) bioluminescent reporter gene. This system can detect acyl-homoserine lactones (acyl-HSL) with acyl chains ranging from six to 12 carbons in length (C_6_ to C_12_ acyl-HSLs).

This strain was used to quantitatively assess the secretion of acyl-HSL molecules by *P. carotovorum* VKM-B1247 in the presence of 2,4-DAPG. Supernatants of *P. carotovorum* cultures treated with 2,4-DAPG were mixed with biosensor *E. coli* MG 1655 pVFR1-lux in 96-well microtiter plates to a final volume of 100 μL. Bioluminescence was recorded as relative light units (RLU) using a Multiscan FL reader (Thermo Scientific, Waltham, MA, USA) every 5 min at 25 °C. The degree of bioluminescence induction was determined as the ratio of the luminescence intensity of the test sample for 90 min to the starting point. Obtained RLU data were recalculated on the biomass value (OD 620) and expressing the result in induction percent comparing to the untreated samples.

### 2.8. Statistical Analysis

The experiments were performed using two independent series with three technical replicates each. The obtained results were statistically manipulated with Origin 2015 (OriginLab Corporation, Northampton, MA, USA) software. 

The Shapiro-Wilk test was used to assess the normal distribution of values. In the presence of a normal distribution, the Student’s *t*-test have been used, indicating the mean and standard deviation (mean ± SD). Differences were considered significant at *p*-value ≤ 0.05.

## 3. Results

### 3.1. Inhibitory Activity of 2,4-DAPG

#### 3.1.1. Spectra of Antimicrobial Activity of 2,4-DAPG 

The minimal bactericidal concentration (MBC) of 2,4-DAPG was determined by measuring the optical density of cell cultures exposed to serially diluted antibiotic concentrations with subsequent seeding on solid medium. The antibiotic was found to be effective against Gram-negative bacteria with MBC concentrations from 48 µg/mL (*Chromobacterium violaceum* ATCC 31532) to 300 µg/mL (*E. coli* K12), while *Pseudomonas* sp. was resistant up to 300 µg/mL (Table 1). Gram-positive bacteria demonstrated more pronounced susceptibility to 2,4-DAPG treatment. *S. aureus* 209P was found to be the most sensitive, with a MBC of 2 µg/mL, while *Enterococcus faecium* ICIS 153 was found to be inhibited at 100 µg/mL (Table 1).

#### 3.1.2. Time-Kill Kinetics

**Figure 1:**
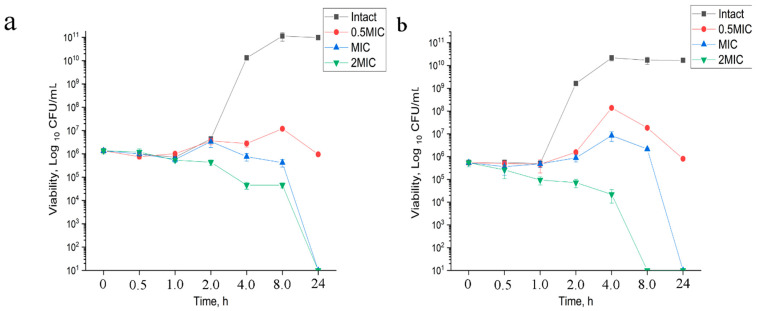
Time kill assay of *E. coli* K12 (**a**) and *S. aureus* 209P (**b**) strains. Killing was tested by incubating bacteria with 2,4-DAPG at the 0.5 MIC, two dilutions above (1×, and 2× MIC) in Mueller–Hinton broth (MHB). Viable cell counts were determined after 0.5, 1, 2, 4, 8 and 24 h of incubation at 37 °C. The growth control had no antibiotic.

The time-kill kinetics profile of 2,4-DAPG against the test organisms *E. coli* K12 and *S. aureus* 209P at high concentrations related to 2 MIC showed a reduction in the number of viable cells over the first 4 h (Figure 1a,b), while complete inhibition has been achieved for 24 h. At the same time, the treatment of bacteria with inhibitory (MIC) and sub-inhibitory (0.5 MIC) concentrations delay bacterial growth for the first 8 h followed by the bactericidal effect on the 24th hour at MIC. In contrast, antimicrobial peptide indolicidin is able to kill bacteria within 1–4 h (Figure S1). 

#### 3.1.3. Fluorescent Investigation of 2,4-DAPG Action on *E. coli* K12

Measurement of membrane permeability based on the quenching of Syto9 fluorescence by propidium iodide, which selectively enters the cell through damaged membranes. If bacterial membranes are permeabilized, propidium iodide penetrates into the cell. What follows is Syto 9 being displaced from nucleic acids, which leads to a decrease in fluorescence intensity in a green region (505–535 nm) of the spectrum. 

**Figure 2:**
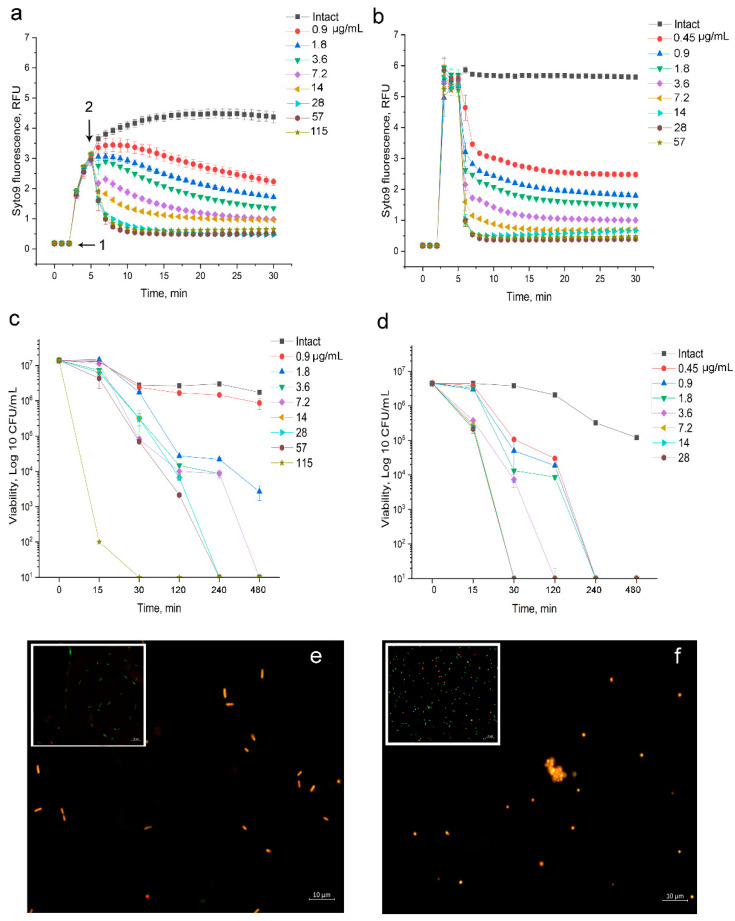
Dynamic of Syto 9 fluorescence measured for 30 min. (**a**) *E. coli* K12 treated with 2,4-DAPG; (**b**) *S. aureus* 209P treated with 2,4-DAPG. Designations: 1—the moment of introduction of the fluorophore; 2—the moment of introduction of 2,4-DAPG;.Time-kill curves of 2,4-DAPG action on *E. coli* K12 (**c**) and *S. aureus* 209 P (**d**) obtained by the agar-drop plate assay. The line plots demonstrated dynamic of bacterial survival as a dependence of 2,4-DAPG concentration and exposure time. Y-axis is a number of viable bacterial cells (Log 10 CFU/mL), while X-axis is time of treatment (minutes); Epifluorescence images of *E. coli* K12 cells (**e**) and *S. aureus* 209P (**f**) treated with 2,4-DAPG. The inset parts of the images showed untreated bacterial populations.

Disturbance of the bacterial barrier structure by 2,4-DAPG was found in Gram-positive bacteria and Gram-negative bacteria. The minimum concentration of 2,4-DAPG that reduces the intensity of Syto9 fluorescence when exposed to *E. coli* K12 cells was 0.9 μg/mL. It should be noted that exposure to 2,4-DAPG in the range 0.9–3.6 μg/mL leads to a relatively slow inhibition of fluorescence, and complete suppression was not achieved during the measurement (Figure 2a).

In turn, the introduction of 2,4-DAPG for 7.2–14 μg/mL led to rapid permeabilization of the barrier structures, which was recorded as decreasing in Syto 9 fluorescence for 3.0–4.5-fold, relative to intact cells (Figure 2a). When 2,4-DAPG was added to the reaction medium from 28 μg/mL and higher, complete suppression of Syto 9 fluorescence within the first 15 min was recorded (Figure 2a).

To understand how the revealed effects affect the viability of bacteria, they have been taken and plated on agar in parallel with the measurement of Syto 9 fluorescence (Figure 2c). It turned out that 2,4-DAPG in a concentration of 0.9 μg/mL does not significantly decrease the number of living cells of *E. coli*. Rapid inhibition of the viability of *E. coli* (within 15 min) was noted when bacteria were exposed to 115 μg/mL (10^7^ to 10^2^ CFU/mL). In turn, treatment of bacteria with 2,4-DAPG taken at 28 and 57 μg/mL reduced the number of viable cells (from 10^7^ to 10^3^ CFU/mL) within 120 min, and complete suppression (10^7^ to 10^1^ CFU/mL) was achieved in 240 min (Figure 2c).

#### 3.1.4. Fluorescent Investigation of 2,4-DAPG Action on *S. aureus* 209P

The methodic based on the measurement of Syto 9 fluorescence kinetic was performed to record the consequence of 2,4-DAPG action on bacteria in a real-time manner. This approach allows distinguishing the mode of action of antibiotics which antimicrobial mechanism has been associated with a disturbance of cell barrier structures but developing in different ways. 

The performed fluorimetry showed two features of the effect of 2,4-DAPG on the *S. aureus* 209P test strain. Firstly, 2,4-DAPG leads to inhibition of Syto9 fluorescence within the first minutes at all used concentrations (Figure 2b). 

Secondly, complete suppression of the Syto9 fluorescence is achieved at an antibiotic concentration of 7.2 μg/mL and higher, while lower concentrations reduce the fluorescence intensity by 3.0–5.5 fold (Figure 2b).

In turn, these results are consistent with the results of the viability assay, which showed a bactericidal effect within 30 min of exposure to 2,4-DAPG taken from 7.2 μg/mL and above (Figure 2d).

As an illustration of the permeabilizing effect of 2,4-DAPG on bacterial cells, fluorescence microscopy of microorganisms stained with the Syto 9/PI.

Initially, populations of *S. aureus* and *E. coli* cells were heterogeneous, consisting primarily of cells with intact membranes and a smaller population with compromised membranes (Figure 2, insertions in e,f). Treatment with 2,4-DAPG for 2 h led to a reduction in the number of the visible bacteria in the population and accumulation of cells glowing red (Figure 2e,f) suggesting membrane permeabilization effect.

#### 3.1.5. Atomic Force Microscopy of Bacterial Cells Treated with 2,4-DAPG

While fluorescent microscopy was able to visualize the membrane-disordering effect of 2,4-DAPG, atomic force microscopy (AFM) was used to provide a more detailed analysis of 2,4-DAPG effect on bacterial morphology. In particular, atomic force microscopy was used to assess the change in bacterial cell morphology over time upon antibiotic treatment.

The most significant changes in cell surface morphology were recorded in the phase mode. Phase-shifting recorded emphasize variations in the sample composition and properties such as viscoelasticity, elasticity, and adhesion [21].

**Figure 3:**
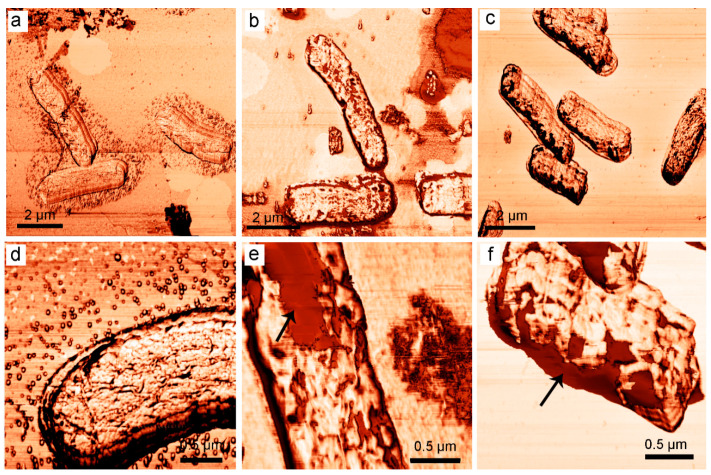
Atomic force microscopy (AFM)-images (phase-mode) of intact *E. coli* K12 bacterial cells (**a**,**d**); treated with 2,4-DAPG and sampled at 15 min (**b**,**e**); 120 min (**c**,**f**). Arrows indicate a disturbance in the bacterial outer membrane, which led to the shifting of phase signal.

Untreated intact *E. coli* K12 bacterial cells have typical a rod-shaped morphology without any cell invaginations or signs of losing of intracellular content (Figure 3a); the outer membrane has been visualized with a homogenous surface architectonic (Figure 3d). The addition of 2,4-DAPG at 115 µg/mL and incubation for 15 min did not lead to significant changes in the size of cells (Figure 3b), but the surface of bacteria was disturbed. It was found the regions of a bacterial surface where obtained AFM-phase signal significantly differs from normal (Figure 3e).

In turn, 2,4-DAPG exposure for 120 min led to a change in cell size, and they are wrinkled (Figure 3c) and the cell surface has been visualized with areas where the phase signal was in more contrast from comparing to intact cells (Figure 3f).

**Figure 4:**
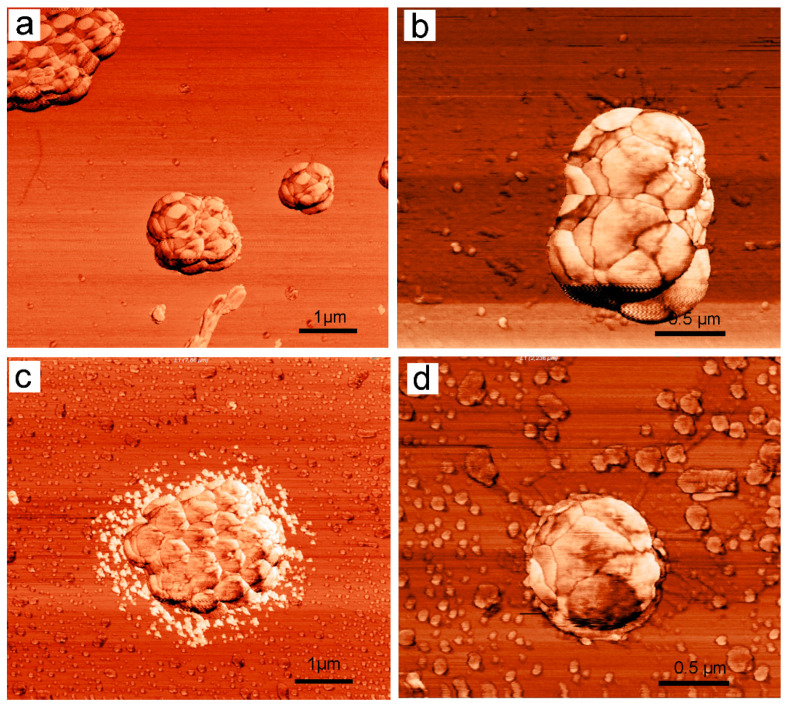
AFM-images (phase-mode) of intact *S. aureus* 209P bacterial cells (**a**,**b**); treated with 2,4-DAPG and sampled at 120 min (**c**,**d**).

Intact *S. aureus* 209P cells were visualized without signs of damage to external structures (Figure 4a). There is no cell debris around the bacterial cells and space between its groups. Detailed AFM-examination of the bacterial surface revealed morphology that is typical of staphylococci (Figure 4b). 

Treatment of *S. aureus* 209P cells with 2,4-DAPG at 28 µg/mL and incubation for 60 min, in general, did not lead to significant changes in cell morphology. However, AFM studies showed the cells with signs of leakage of intracellular contents (Figure 4c). In particular, around the cells, a granular material was visualized in abundance (Figure 4a). The phase AFM-signal that was obtained by scanning the bacterial surface did not differ from that of intact bacteria (Figure 4d). 

#### 3.1.6. Anti-Biofilm Action of 2,4-DAPG 

To determine the effectiveness of 2,4-DAPG against biofilms, *S. aureus* 209P and *E. coli* K12 cultures were grown for 48 h to obtain a mature biofilm. 2,3,5-triphenyl-tetrazolium chloride solution was added to the bacterial biofilms. Results were analyzed by spectrophotometer to determine the metabolic activity of living bacterial cells.

**Figure 5:**
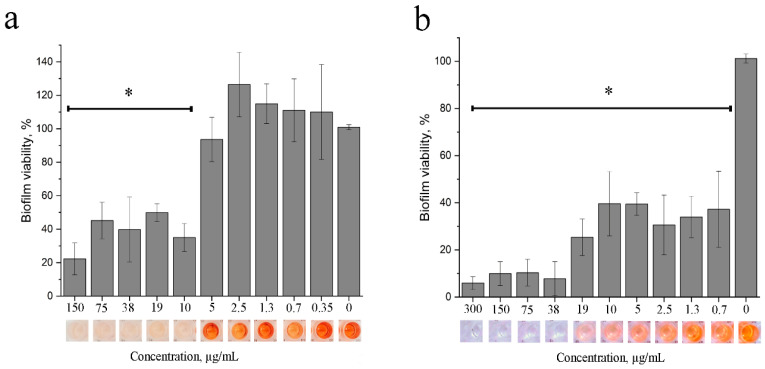
The ability of 2,4-DAPG to inhibit the metabolic activity of *S. aureus* 209P (**a**) or *E. coli* K12 (**b**) bacterial cells are embedded into the biofilm. Metabolic status of the bacterial biofilms was assessed by measurements of formazan absorbance and calculation of percentages relative to intact sample. * Differences were considered significant at *p*-value ≤ 0.05.

Treatment with 2,4-DAPG at concentrations of 10 µg/mL or more reduced the metabolic activity of the biofilm embedded *S. aureus* cells by 50% compared with the control group (Figure 5a). However, complete inhibition was not achieved even at 150 µg/mL. 

Treatment of *E. coli* biofilms with 2,4-DAPG at concentrations of 0.7–19 µg/mL halved the metabolically active bacteria, while 90% inhibition was achieved at concentrations of 38 µg/mL (Figure 5b). 

### 3.2. Sub-Inhibitory Activity of 2,4-DAPG 

#### 3.2.1. SOS-Response and Protein Damage of Bacteria under the 2,4-DAPG Action

**Figure 6:**
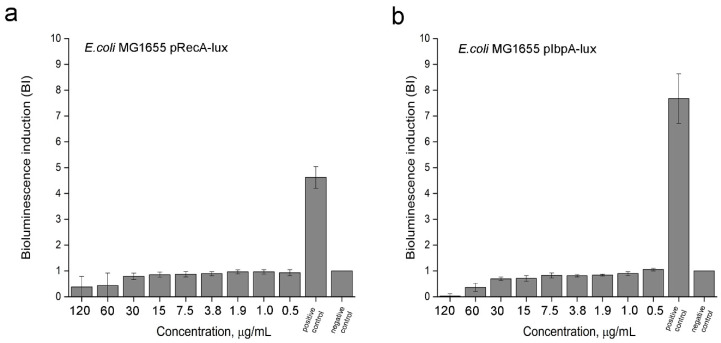
Induction of bioluminescence of the reporter strains under the treatment with various concentration of 2,4-DAPG. (**a**) Assay on SOS-response, where positive control is the sample treated with inductor (fluoroquinolone antibiotic norfloxacin); negative control is the sample without inductor. (**b**) Assay on protein damage, where positive control is the sample treated with inductor (4.8% water solution of ethanol); negative control is the sample without inductor.

The effect of 2,4-DAPG on intracellular targets, including DNA-replication machinery and protein damage, was investigated using highly sensitive bacterial biosensor strains expressing bioluminescence genes under the promoters of the genes of SOS-response and heat shock proteins. The treatment with 2,4-DAPG for 12.5 µg/mL did not lead to induction of bioluminescence in *E. coli* MG 1655 pRecA-lux which are sensitive to fluoroquinolone antibiotics and other genotoxic substances (Figure 6a). The similar lack of the biosensor response to 2,4-DAPG exposure was recorded using *E. coli* MG 1655 pIbpA-lux whose bioluminescence induction is under the control of the promoters of the heat shock genes *ibpA* (Figure 6b). Thus, 2,4-DAPG at subinhibitory concentrations does not affect DNA replication or protein synthesis in bacterial cells.

#### 3.2.2. Influence of 2,4-DAPG on the Production of Acyl-Homoserine Lactone Molecules by *Pectobacterium carotovorum*

Production of acyl-homoserine lactone autoinducers (AI) by *P. carotovorum* began immediately following the beginning of log-phase growth, with a decrease in AI amount as cell growth became stationary. 2,4-DAPG was added to *P. carotovorum* populations in the beginning of log-phase and growth monitoring continued by a spectrophotometer. Three hours later, the amount of AI produced in culture media of pectobacteria was analyzed by recombinant *E. coli* MG 1655 pVFR1-lux, which contains the plasmid pVFR1 carrying a 1-kb DNA fragment of *A. fischeri* including *luxR* and P*l*/P*r* promoters along with a *luxCDABE* cassette of *Photorhabdus luminescens* [19]. LuxR is a quorum-sensitive transcriptional regulator of the *luxICDABEG* operon. After binding to the autoinducer (AI), the LuxR protein acquires the ability to form a complex with the *lux* box and activate the transcription of the *luxICDABEG* operon. Bioluminescence response of *E. coli* MG 1655 pVFR1-lux is proportional to the amount of acyl-homoserine lactones in the medium. 

**Figure 7:**
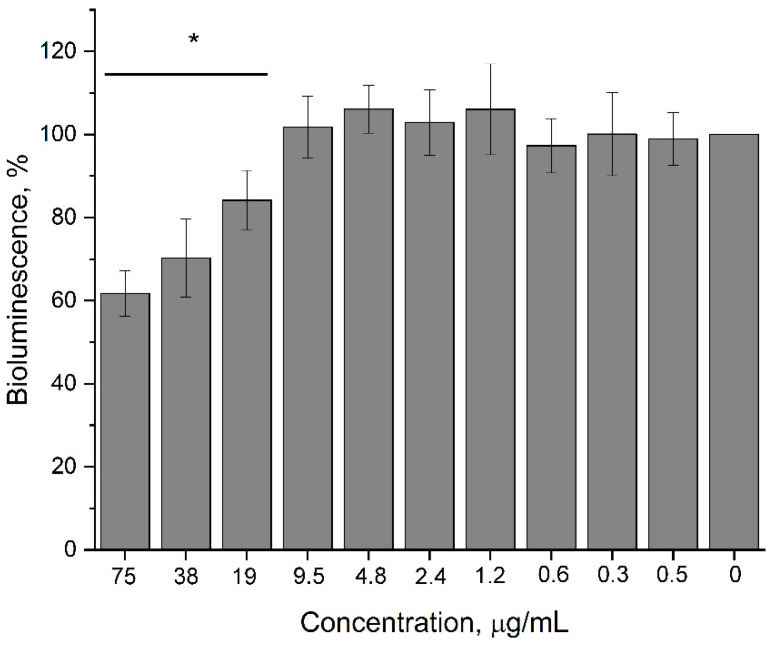
Influence of 2,4-DAPG on the production of acyl-homoserine lactones (acyl-HSL) autoinducers by *P. carotovorum* VKM-B-1247. Bioluminescence induction of the biosensor *E. coli* MG 1655 pVFR1-lux is proportional to the amount of acyl-HSL in the tested culture medium. Y-axis is bioluminescence induction as a percent from that of the untreated sample; X-axis is 2,4-DAPG concentrations used. * Differences were considered significant at *p*-value ≤ 0.05.

Increasing 2,4-DAPG concentrations reduced AI production by *P. carotovorum* that was determined by comparing bioluminescence induction of the biosensor supplemented by culture medium of treated and intact pectobacteria. Minimally, 2,4-DAPG concentrations of 19 µg/mL lowered the amount of AI in the medium by 15.9 ± 7.2% relative to the intact sample (*p* < 0.05). Increasing the concentration of antibiotic in the medium to 38 and 75 µg/mL reduced the amount of autoinducer by 29.8 ± 9.4 and 38.3 ± 5.5%, respectively (Figure 7). However, antibiotic concentrations of 38 µg/mL began to affect the growth of *P. carotovorum*.

#### 3.2.3. Impact of 2,4-DAPG on Quorum Sensing (QS)-Dependent Bioluminescence

In order to understand whether the QS-inhibitory effect of 2,4-DAPG was a manifestation of toxic stress or a dysfunction of the QS-regulatory system (in particularly through abolishing of AI perception by the LuxR receptor), the direct effect of the antibiotic on bioluminescent biosensors was investigated. Two biosensors were taken, including the *E. coli* MG1655 pVFR1-lux containing the LuxR receptor, whose bioluminescence was dependent upon exogenous autoinducers. *E. coli* MG 1655 pRecA-lux was also used, whose bioluminescence was independent of the quorum-sensing system.

**Figure 8:**
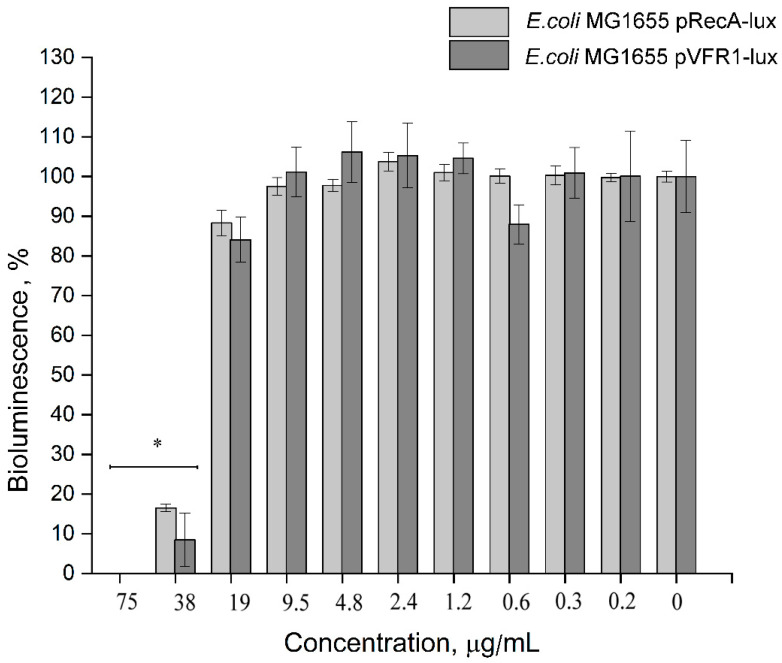
Inhibition of bioluminescence of *E. coli* MG 1655 pVFR1-lux (QS-dependent) (dark gray column) and *E. coli* MG 1655 pRecA-lux (QS-independent) (light gray column) under the treatment with 2,4-DAPG. * Differences were considered significant at *p*-value ≤ 0.05.

It was found that treatment of the *E. coli* MG 1655 pVFR1-lux sensor with 38 μg/mL and higher completely inhibited the bioluminescent response. Bioluminescence of the control biosensor *E. coli* MG 1655 pRecA-lux was inhibited at the same concentration range 38–75 μg/mL (Figure 8). It should be noted that even maximum concentrations of 2,4-DAPG did not reduce the number of CFU of the biosensor strains (Figure S4). 2,4-DAPG was found to affect some metabolic processes in bacteria (bioluminescence), but the QS-quenching effect most likely does not relate to ligand-receptor (C6-HSL-LuxR) interaction.

## 4. Discussion

2,4-Diacetylphloroglucinol belongs to a large group of naturally derived phenolic antimicrobial substances, which possess a well-documented role in the protection of plants. Both plants and microorganisms are known to produce phenolic compounds for self-defense. Among microorganisms, polyphenols such as 2,4-diacetylphloroglucinol and the antibiotic pyoluteorin are one of the most known.

In this work, we qualitatively and quantitatively evaluated the spectrum of antimicrobial activity of 2,4-diacetylphloroglucinol. The spectrum of antimicrobial action of polyphenolic substances is quite extensive, but bacteria with a Gram-negative type of cell wall are more resistant to phenolic compounds compared to Gram-positive bacteria, likely due to the substance targeting of the bacterial cell envelope [22].

Barrier structures of bacterial cells are one of the main targets of the action of polyphenols [23].

2,4-DAPG as a polyphenolic amphipathic molecule could realize its antimicrobial action due to hydrophobic interactions with biological membranes. As noted in the work of Gong, et al. [24], lipophilicity seems to be a principal factor to influence the antibacterial activity of the 2,4-DAPG analogs.

2,4-DAPG mediated destruction of bacterial cells has a number of features. First, permeabilization occurs instantly, which is similar to the action of membrane-disturbing agents determined earlier [25]. Second, disturbance in barrier structures of bacterial cells has been accomplished by reducing its viability that was similar in time and concentrations. The observed difference in the permeabilisation kinetics can be explained by fundamental differences between *S. aureus* and *E. coli* in the organization of their cell walls.

The use of atomic force microscopy allows for the visualization of the interaction of antibiotics with target cells in ways that are inaccessible to other types of microscopy. The phase contrast can be explained by the energy dissipation of the vibrating cantilever due to the adhesive interactions between the AFM tip and the sample surface, as well as by the local viscoelastic properties of the sample [26]. Survey AFM scans demonstrate that the surface of bacteria undergoes some morphological changes upon contact with 2,4-DAPG molecules. Rendered areas with increased phase contrast are likely to have a different viscosity comparing to unaffected regions. Somewhat like images were collected with bacterial capsules where dehydration/hardening of the capsule material led to a decrease in the phase contrast between the mica and the capsules [26]. 

Mechanistic studies of the antimicrobial action of phenolic substances has indicated the multi-target nature of their antibacterial effect [27]. Numerous polyphenols have shown inhibitory activity against DNA gyrase [28]. Flavonoids have demonstrated the ability to reduce membrane fluidity [29]. Some phenolic compounds are also able to interfere with peptidoglycan biosynthesis [30]. In this work, the use of biosensors sensitive to DNA-damage (p*RecA-lux*) and protein-denaturing (p*IbpA-lux*) agents did not record any effects of 2,4-DAPG on these targets. Characteristic features of 2,4-DAPG include its rapidly permeabilizing effect and characteristic morphological changes in bacterial cells.

**Figure 9:**
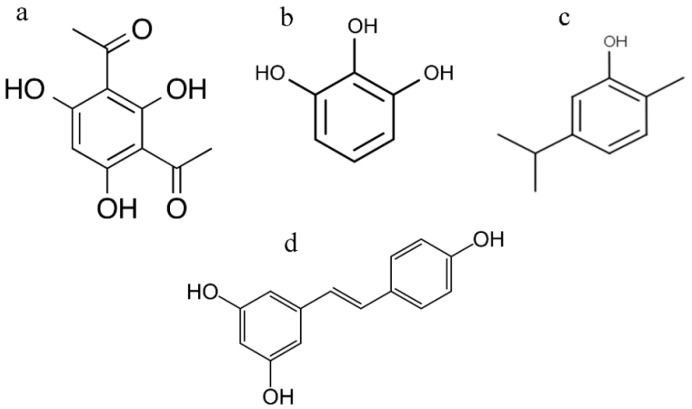
Chemical structure of 2,4-diacetylphloroglucinol (**a**), pyrogallol (**b**), carvacrol (**c**), resveratrol (**d**).

2,4-Diacetylphloroglucinol also demonstrated a subinhibitory effect on quorum-sensing (QS) mediated behavior of bacterial cells. The possibility that 2,4-DAPG is capable of inhibiting the processes associated with QS follows indirectly from the structural similarities with known QS-inhibitors such as eugenol, carvacrol, pyrogallol, and resveratrol (Figure 9a–d) [31,32,33]. Moreover, it was shown that phloroglucinol is capable of functioning as a chemical messenger regulating gene expression in *Pseudomonas* spp. [34]. However, experiments using *C. violaceum* have revealed that phloroglucinol did not interfere with QS-regulated violacein production [35].

*P. carotovorum* VKM-B-1247 was chosen as model organism to evaluate the effects of 2,4-diacetylphlorglucnol on the quorum sensing-related physiology of bacteria. *P. carotovorum* is a phytopathogenic strain whose virulence is controlled by a quorum-sensing mechanism [36,37]. The introduction of 2,4-diacetylphloroglucinol was during the middle part of logarithmic growth, followed by a three-hour incubation with bacteria inhibited acyl-HSL production in pectobacteria.

2,4-DAPG was tested on the *E. coli* MG 1655 pVFR1-lux strain in the presence of exogenous C6-HSL molecules to determine whether the inhibition of autoinducers production was a consequence of the blockage of AI receptor. It turned out that 2,4-DAPG inhibits the bioluminescence of the biosensor, without affecting cell viability. To prevent a false-positive result, *E. coli* MG 1655 pRecA-lux strain, characterized by some constitutive level of bioluminescence, was chosen as a negative control [38]. 2,4-DAPG was found to inhibit the bioluminescence of the *E. coli* pRecA-lux strain (QS-independent) at the same concentration range as the bioluminescence of *E. coli* pVFR1-lux (QS-dependent). This indicates that the suppression of the synthesis of QS-autoinducers of pectobacteria does not occur during the reception of signaling molecules.

Despite this, the possibility that subinhibitory concentrations of 2,4-diacetylphloroglucinol were able to impact the normal functioning of QS-regulated processes in some other way cannot be excluded. It is believed that the subinhibitory effects of 2,4-DAPG are dependent on its ability to damage cell membranes. It is known that many receptors of two- and three-component regulatory systems of Gram-negative and Gram-positive bacteria are closely associated with cell membranes. The slightest disturbances in the structure of cell membranes can signal for the rearrangement of bacterial cell physiology [39]. This could explain the results of Powers et al. [5] which are found that 2,4-DAPG inhibited expression of genes involved in biofilm formation of *Bacillus subtilis*.

In this work we evaluated the inhibitory and subinhibitory activity of 2,4-diacetylphloroglucinol, a molecule that is considered to be a natural antibiotic which regulates interspecies relationships in plant ecosystems. Results obtained showed the ability of 2,4-DAPG to affect metabolic activity of bacteria with various structural organization.

## Figures and Tables

**Table 1:** Spectra of antimicrobial activity of 2,4-DAPG (2,4-diacetylphloroglucinol).

Type of Cell Wall	Strains	MIC, µg/mL	MBC, µg/mL
Gram-positive bacteria	*Staphylococcus aureus* 209 P	2.0	2.0
	*Clavibacter michiganensis* VKM AS-1405	25	25
	*Enterococcus faecium* ICIS 153	50	100
Gram-negative bacteria	*Pectobacterium caratovorum* VKM-B1247	90	90
	*Chromobacterium violaceum* ATCC 31532	24	48
	*Pseudomonas savastanoi* VKM-1546	>300	>300
	*Escherichia coli* K12	300	300
	*Pseudomonas aeruginosa* ATCC 28753	>300	>300

## Data Availability

Author elects to not share data.

## Supplementary Materials

The following are available online at https://www.mdpi.com/2218-273X/11/1/13/s1, Figure S1: Time-kill assay of indolicidin with *E. coli* K12 and *S. aureus* 209P strains, Figure S2: AFM-images (MAG-mode) of intact *E. coli* K12 bacterial cells, and treated with 2,4-DAPG (sampled at 15 min, 120 min), Figure S3: AFM-images (MAG-mode) of intact *S. aureus* 209P bacterial cells treated with 2,4-DAPG (sampled at 120 min), Figure S4: Impact of 2,4-DAPG on the viability of biosensors, that was assessed at the end-point of co-incubation.
