# Supplementary material for: Bacterial-Derived Plant Protection Metabolite 2,4-Diacetylphloroglucinol: Effects on Bacterial Cells at Inhibitory and Subinhibitory Concentrations
Source: Biomolecules. 2020 Dec 25;11(1):13. doi: 10.3390/biom11010013 (PMC7823703; doi:10.3390/biom11010013)
Supplement: Supplementary file 1 [file biomolecules-11-00013-s001.pdf]

Bacterial-Derived Plant Protection Metabolite 2,4-Diacetylphloroglucinol: Effects on Bacterial Cells at Inhibitory and Subinhibitory Concentrations

William T. Julian [1], Anastasia V. Vasilchenko [2], Daniil D. Shpindyuk [1], Darya V. Poshvina [1] and Alexey S. Vasilchenko [1,*]

[1] Laboratory of Antimicrobial Resistance, Institute of Environmental and Agricultural Biology (X-BIO), Tyumen State University, Tyumen, Russia

[2] All-Russian Institute of Plant Protection, St. Petersburg-Pushkin, Russia

*avasilchenko@gmail.com

Key words: 2,4-diacetylphloroglucinol, plant protection, membrane permeabilization, subinhibitory effects, quorum sensing, *Pectobacterium carotovorum*.

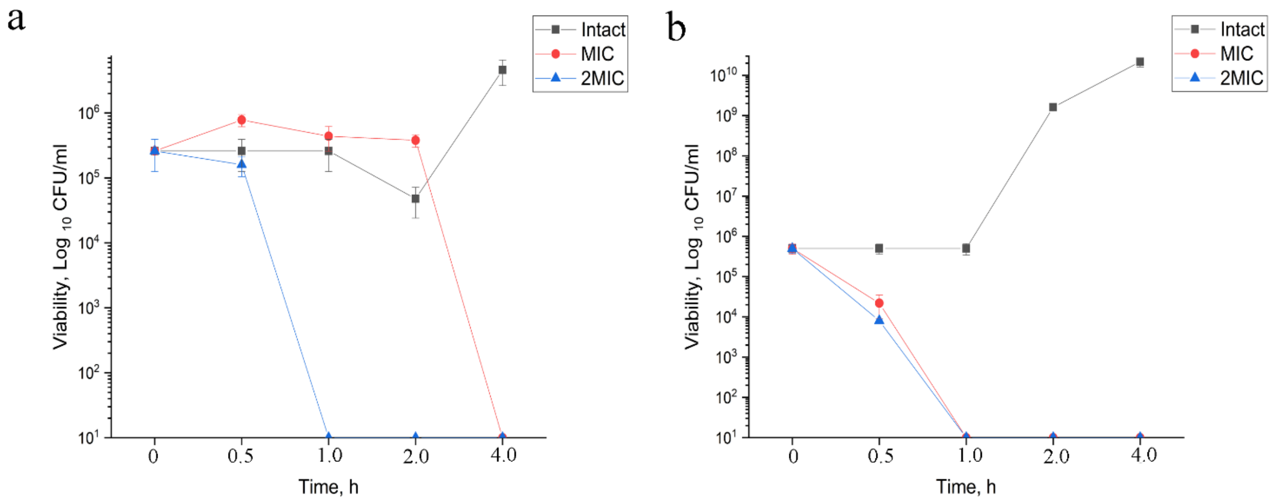

Figure S1. Time kill assay of *E.coli* K12 (**a**) and *S.aureus* 209P (**b**) strains. Killing was tested by incubating bacteria with indolicidin at the MIC, two dilutions above (2MIC) in MHB. Viable cell counts were determined after 0.5, 1, 2, 4 h of incubation at 37 °C. The growth control had no antibiotic.

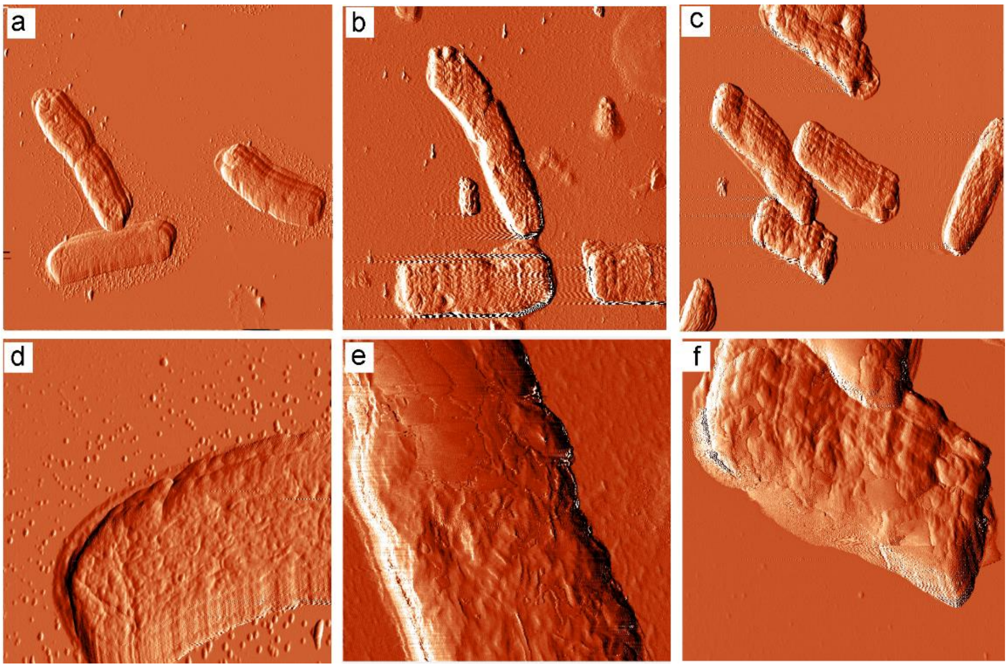

Figure S2. AFM-images (MAG-mode) of intact *E.coli* K12 bacterial cells (**a**, **d**); treated with 2,4-DAPG and sampled at 15 min (**b**, **e**); 120 min (**c**, **f**).

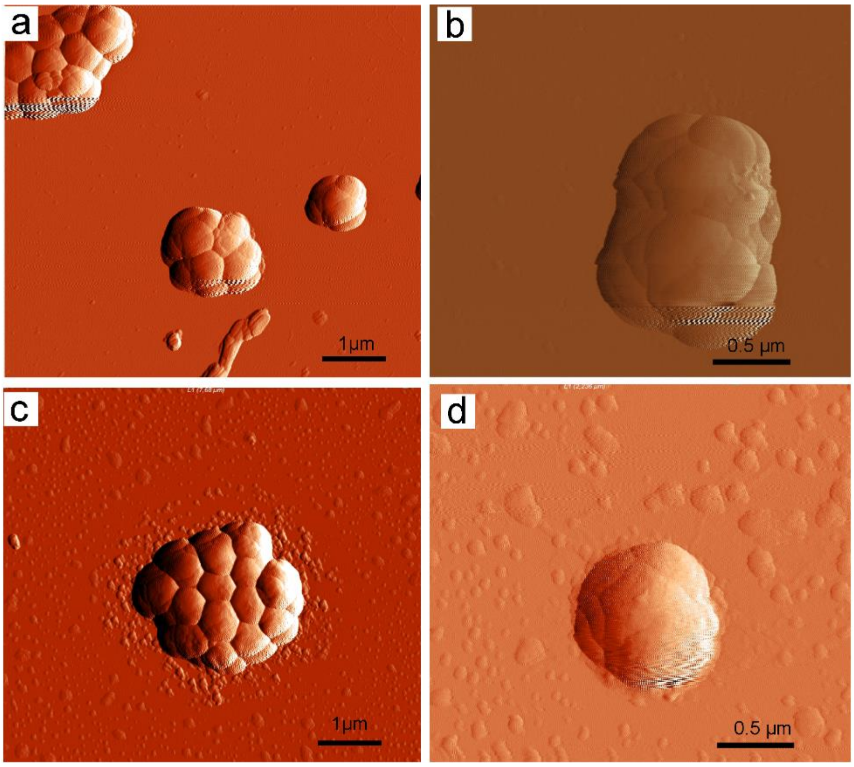

Figure S3. AFM-images (MAG-mode) of intact *S.aureus* 209P bacterial cells (**a**, **b**); treated with 2,4-DAPG and sampled at 120 min (**c**, **d**).

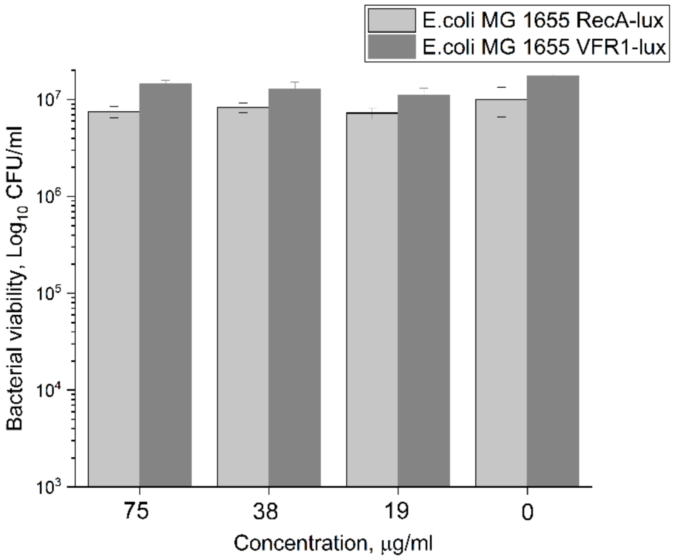

Figure S4. Impact of 2,4-DAPG on the viability of biosensors, that was assessed at the end-point of co-incubation.
